# Toxicogenomic Screening of Replacements for Di(2-Ethylhexyl) Phthalate (DEHP) Using the Immortalized TM4 Sertoli Cell Line

**DOI:** 10.1371/journal.pone.0138421

**Published:** 2015-10-07

**Authors:** Thomas C. Nardelli, Hanno C. Erythropel, Bernard Robaire

**Affiliations:** 1 Department of Pharmacology and Therapeutics, McGill University, Montreal, Quebec, Canada; 2 Department of Chemical Engineering, McGill University, Montreal, Quebec, Canada; 3 Department of Obstetrics and Gynecology, McGill University, Montreal, QC, Canada; University of Nevada School of Medicine, UNITED STATES

## Abstract

Phthalate plasticizers such as di(2-ethylhexyl) phthalate (DEHP) are being phased out of many consumer products because of their endocrine disrupting properties and their ubiquitous presence in the environment. The concerns raised from the use of phthalates have prompted consumers, government, and industry to find alternative plasticizers that are safe, biodegradable, and have the versatility for multiple commercial applications. We examined the toxicogenomic profile of mono(2-ethylhexyl) phthalate (MEHP, the active metabolite of DEHP), the commercial plasticizer diisononyl cyclohexane-1,2-dicarboxylate (DINCH), and three recently proposed plasticizers: 1,4-butanediol dibenzoate (BDB), dioctyl succinate (DOS), and dioctyl maleate (DOM), using the immortalized TM4 Sertoli cell line. Results of gene expression studies revealed that DOS and BDB clustered with control samples while MEHP, DINCH and DOM were distributed far away from the control-DOS-BDB cluster, as determined by principle component analysis. While no significant changes in gene expression were found after treatment with BDB and DOS, treatment with MEHP, DINCH and DOM resulted in many differentially expressed genes. MEHP upregulated genes downstream of PPAR and targeted pathways of cholesterol biosynthesis without modulating the expression of PPAR’s themselves. DOM upregulated genes involved in glutathione stress response, DNA repair, and cholesterol biosynthesis. Treatment with DINCH resulted in altered expression of a large number of genes involved in major signal transduction pathways including ERK/MAPK and Rho signalling. These data suggest DOS and BDB may be safer alternatives to DEHP/MEHP than DOM or the commercial alternative DINCH.

## Introduction

Plasticizers are compounds that are added to brittle polymers, such as poly(vinyl chloride) (PVC), to increase their flexibility and malleability. It is estimated that in 2006, total plasticizer production totalled 5.8 million metric tons, of which phthalates made up 75% of production [1]. Di-(2-ethylhexyl) phthalate (DEHP) is the most commonly used phthalate for plasticizing PVC. It is able to plasticize PVC because it contains both polar moieties that ensure compatibility with the polymer, and non-polar moieties that are able to disrupt the polar interactions between adjacent PVC polymer chains [2]. During manufacturing, it is common for PVC products to contain up to 40% plasticizer (such as DEHP) by weight, but the final amount depends on the desired physical properties of a plastic [3]. One caveat of using DEHP is that it does not form covalent bonds with PVC; therefore, plasticizers can leach out over time into the environment ultimately resulting in human exposure [4, 5].

Although human exposure to DEHP is mainly due to leaching from PVC, it and other lower molecular weight phthalates can also be found in cosmetics where they are used as emulsifiers and solvents [6]. As a result of ubiquitous exposure, phthalates and their metabolites are readily found in urine, breast milk and serum [7]. Recent restrictions on the use of six phthalate compounds in selected products have been implemented to reduce the phthalate burden of neonates [8–10] as children who would chew phthalate containing plastics while teething or those who require extensive perinatal care had a phthalate burden as high as one to two orders of magnitude greater than adults [6, 11–13]. Combined with a smaller body mass and impaired metabolic pathways, neonates are one of the most vulnerable demographics to the effects of phthalates [14, 15].

Phthalates have well documented anti-androgenic effects [16], but the mechanism by which they exert these effects is still not fully understood. Pathways involving members of the peroxisome proliferator-activated receptor (PPAR) family have also been identified as potential mediators of phthalate toxicity. MEHP can activate several PPAR isoforms [17] and PPAR-alpha null Sv/129 mice have a milder phenotype than wild-type following treatment with DEHP [18]. In addition to direct pertubation of PPAR signalling, PPAR dysregulation can alter other nuclear receptor signalling pathways, such as the retinoic acid and thyroid hormone signalling pathways, by sequestering endogenous heterodimer binding partners or by biasing heterodimer formation [19, 20]. As nuclear receptor signalling via these and other nuclear pathways is important for proper gonadal development and spermatogenesis [21, 22], it has been proposed that phthalate exposure may in part be responsible for “testicular dysgenesis syndrome"; an umbrella term for clinical presentations of cryptorchidism, hypospadia, testicular cancer, and decreased sperm production that are believed to be caused by a common developmental etiology [23].

Phthalate reproductive toxicity is complex as multiple cell types have been proposed as targets [24]. The Sertoli cell is considered to be a mediator of phthalate toxicity as it has a critical role in gonadal sex-determination, testicular development, and spermatogenesis [25]. Several strains of immortalized Sertoli cells have been derived for *in vitro* use to simplify complex biological systems involving multiple cell types to pinpoint cell specific mediated toxicity. The 15P-1 Sertoli cell line is derived from transgenic adult mice expressing the large T protein of polyoma virus [26]. 15P-1 maintains the expression of Wilms’ tumor and Steel genes and can support meiotic differentiation of germ cells [26]. The MSC-1 cell line is derived from adult mice using small and large T-antigens from the SV40 virus. While it does not express the follicle stimulating hormone receptor, it maintains characteristic expression of transferrin, clusterin, and inhibin βb [27]. The TM4 Sertoli cell line is derived from 11–13 day old mice and resembles immature Sertoli cells. It is well characterized, has not been transformed, is not tumorigenic, and maintains many important aspects of Sertoli cell physiology. It is a particularly good model for endocrine disruption studies as it maintains the ability to respond to FSH stimulation, and expresses both androgen and estrogen receptors [28–30].

High-throughput *in vitro* and *in silico* methods combined with cell culture methods have provided the tools necessary to screen and identify deleterious compounds in an effective and cost-efficient manner [31]. Previous *in vitro* studies have shown MEHP can decrease pyruvate and ATP production while increasing reserves of intracellular lipids in Sertoli cells [25]. Phthalate monoesters can also disrupt Sertoli-germ cell cross-talk and promote germ cell apoptosis via FASL/FAS signalling pathway by increasing MMP2 activity and downstream cleavage of TNFα [32, 33]. Furthermore, phthalates can cause precocious release of germ cells into the lumen of the seminiferous tubules by disrupting ectopic specialization formation and other cytoskeletal components of Sertoli cells involved in germ cell transit [34].

**Fig 1:**
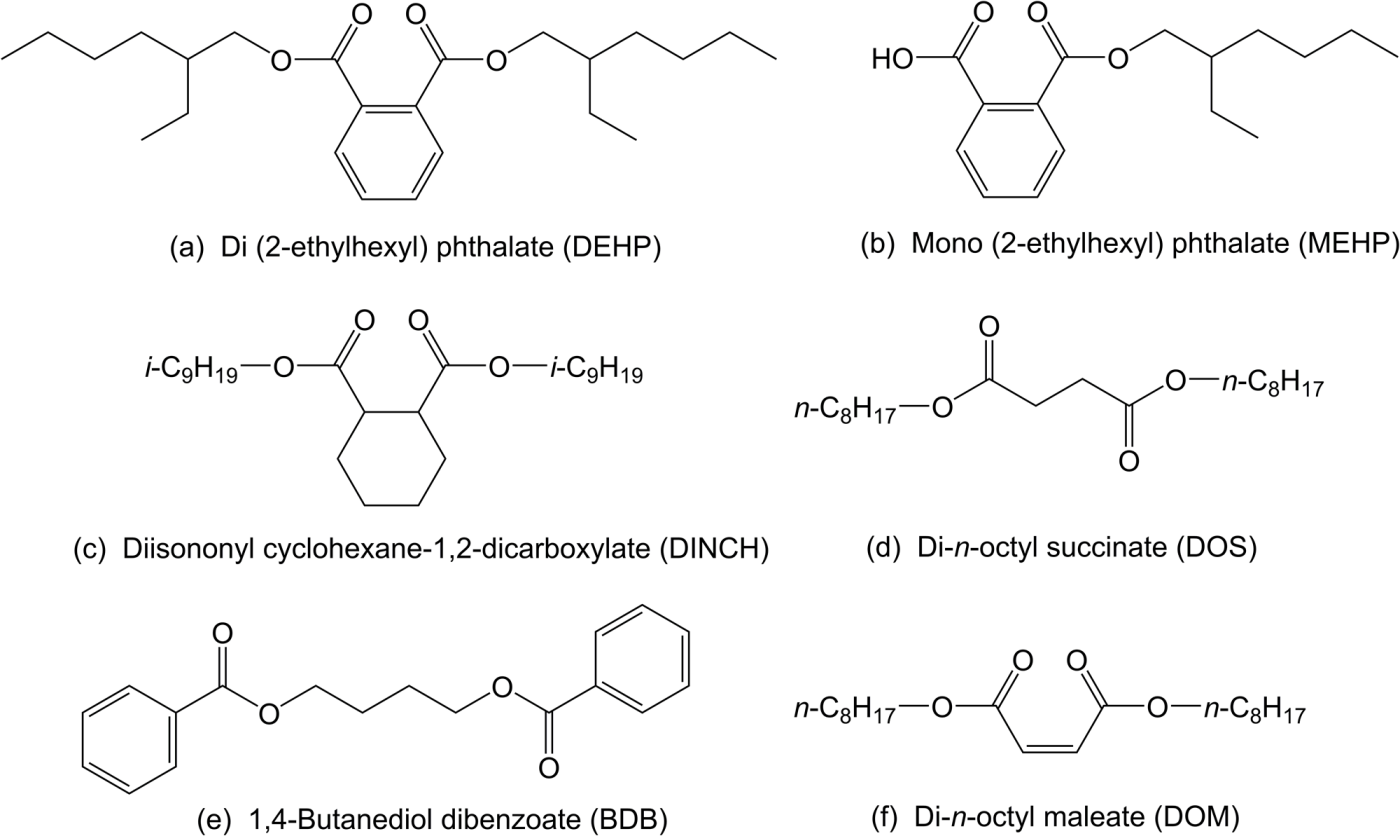
Chemical structures. The phthalate plasticizer DEHP (a) and its main bioactive metabolite MEHP (b), a current commercial replacement DINCH (c), and three alternative plasticizers: DOS (d), BDB (e), and DOM (f).

A variety of alternative plasticizers have been proposed as possible replacements for DEHP, but there is a lack of data regarding their safety. S1 Table provides a list of several alternatives, and Fig 1 shows the compounds subsequently chosen for this study. Diisononyl cyclohexane-1,2-dicarboxylate (DINCH) is a commercial plasticizer marketed for applications involving close human contact [35]; however, there are very few peer-reviewed studies on its effects in biological systems [36]. Dioctyl succinate (DOS) and dioctyl maleate (DOM) are part of a series of proposed replacement plasticizers that structurally resemble phthalates [37–39]. Second generation dibenzoate plasticizers, such as 1,4-butanediol dibenzoate (BDB), have also been proposed to address concerns raised by commercial diethylene- and dipropylene-glycol dibenzoate plasticizers as the latter two compounds lead to the formation of persistent toxic metabolites in the presence of common soil microorganisms [40, 41].

With the decrease in cost and increased sensitivity, microarray experiments have become effective for high-throughput xenobiotic screening [42]. By generating a toxicogenomic fingerprint, a novel compound can be compared to a database of known toxicants to predict unwanted toxicity [42, 43]. A similar toxicogenomic strategy using fetal testes from pups who were exposed to a series of phthalates during gestation successfully segregated developmentally toxic phthalates from inert ones with good correlations to previous findings in the literature based solely on differential gene expression [44]. Furthermore, phthalates deemed developmentally toxic targeted similar genes regulating steroidogenesis, lipid and cholesterol homeostasis, and several other important pathways [44]. Similarly, structure-function analysis and read-across has been used to identify families of compounds that share common mechanisms of toxicity [45]. Taken together, these strategies can be used to predict toxicity, prioritize screening, reduce development costs, and minimize the use of animals in toxicity screening of novel plasticizers before their commercial use [46, 47].

In this study, the toxicity of MEHP, the bioactive metabolite of DEHP, an alternative commercial plasticizer (DINCH), and three novel plasticizers (DOM, BDB and DOS) were assessed using cell viability and toxicogenomic methods in immortalized Sertoli cell lines.

## Materials & Methods

### Source of Chemicals

DEHP was purchased from Sigma-Aldrich Corporation (Cat#80030, St. Louis, MO), MEHP was purchased from Wako Pure Chemical Industries (Cat#323–65643, Osaka, Japan), DINCH was supplied by BASF Canada (Mississauga, ON). DOM, DOS, and BDB were synthesized as previously described [37–39]. In addition to these compounds, analogues shown in S1 Table were also tested for effects on cell viability in three immortalized cell lines. The maleates, succinates, fumarates were chosen due to their structural similarity to DEHP [37–39] while the dibenzoates are structurally similar to diethylene glycol dibenzoate plasticizers [40, 48]. These compounds were either synthesized in-house or purchased as indicated in S1 Table.

### Cell Cultures

MSC-1 (donated by Dr Robert Viger, Centre hospitalier universitaire de Québec, Charlesbourg, QC, cell line originally derived and characterized in [27]) and TM4 (CRL1715, ATCC, Manassas, VA) cells were cultured at 37°C with 5% CO_2_ in either DMEM supplemented with 10% FBS, and 0.5% Penicillin-Streptomycin (P/S) or DMEM:F12 (ATCC) supplemented with 2.5% FBS, 5% Horse Serum, and 0.5% P/S respectively. 15P-1 cells (CRL-2618, ATCC, Manassas, VA) were cultured at 32°C with 5% CO_2_ in DMEM supplemented with 5% FBS, 1% sodium pyruvate, and 0.5% P/S. All cell culture reagents were purchased from Wisent (St-Bruno, QC) unless otherwise indicated.

### Cell Viability Assay

The MTT assay is an indirect measurement of cell viability that measures the conversion of 3-(4,5-dimethylthiazol-2-yl)-2,5-diphenyltetrazolium bromide (MTT) to a purple formazan. This conversion occurs primarily in the mitochondria of living cells. Cells were seeded in Costar 96-well plates (Corning, Tewksbury, MA) and allowed to adhere for 24hrs. The culture media was aspirated and replaced with media containing either vehicle or plasticizer at concentrations ranging from 10^-8^ to 10^-4^M in 10 fold increments. Dimethyl sulfoxide (DMSO, Sigma-Aldrich, Oakville, ON) was used as vehicle at 0.4% at all concentrations except 10^-4^M where 1% DMSO was used; all values were compared to their respective vehicle control (S1 and S2 Figs). After 44hrs, 50μg of MTT (Millipore, Temecula, CA) was dissolved in 1x phosphate buffered saline (PBS; 1.71M NaCl, 0.03M KCl, 0.06M Na_2_HPO_4_·2H_2_O, 0.02M KH_2_PO_4_) and was added to each well for an additional 4hr incubation. Following incubation, the cell culture medium was carefully aspirated and the MTT crystals were dissolved using 100μL DMSO. The optical density was measured using a SpectraMax Plus 384 (Molecular Devices, Sunnyvale, CA) spectrophotometer. The absorbance at 620nm was subtracted from 570nm to correct for background.

### RNA Extraction, Quantification and Purity

TM4 cells were seeded in six-well plates containing 250,000 cells in each well and allowed to adhere for 24 hours. The culture medium was aspirated and fresh media with 10^-4^M treatment (DOS, BDB, MEHP, DOM, and DINCH) or vehicle (1.0% DMSO) were added and allowed to incubate for 48hrs.

Following incubation, the RNeasy Plus Mini Kit (Qiagen, Toronto, ON) was used for RNA extraction. The culture medium was aspirated and 600μL Buffer RLT supplemented with 6μL β-mercaptoethanol (Sigma-Aldrich, Oakville, ON) was added to each well. Samples were pipetted several times to mechanically disrupt the cells and stored at -80°C for future extraction. On the day of extraction, lysates were further homogenized using QIAShredder columns (Qiagen). RNA was extracted from the flow-through as per the manufacturer's instructions.

RNA purity was determined using a NanoDrop 2000 spectrophotometer (ThermoFisher, Waltham, MA) to determine 260/280 and 260/230 ratios in order to ensure samples did not contain DNA, and were free of chemical contaminants used during the extraction process that may affect downstream applications. RNA integrity, quantity, and purity were further analysed using the RNA 6000 Nano kit (Agilent Technologies, Santa Clara, CA) as per manufacturer’s instructions. All samples had integrity (RIN) values > 9.8 as determined using the 2100 Bioanalyzer (Agilent Technologies).

### Gene Expression Microarray

RNA was converted to cRNA and labeled with Cy3 using the Low Input Quick Amp Labeling Kit (Agilent Technologies) following the manufacture’s protocol starting with 100ng of RNA. Only samples yielding more than 1.65μg and having a specific activity greater than 9.0 pmol Cy3/μg cRNA were hybridized to microarray chips. This was determined using the NanoDrop 2000c (ThermoFisher). cRNA was hybridized to SurePrint G3 Mouse GE 8x60K Microarrays (Agilent Technologies) for 17 hours at 65°C. Microarray chips were scanned using the SureScan G2600D Microarray Scanner (Agilent Technologies). Probe intensities were converted to numerical values using Feature Extraction ver. 11.5.1.1 (Agilent Technologies) software with protocol GE1_1105_Oct12 and grid 028005_D_F_20110614. Probe values were imported into GeneSpring 12.6.1 GA-PA (Agilent Technologies) and normalized using percentile shift normalization to the 75^th^ percentile with a baseline transformation to the median of all samples. Each treatment was analysed independently of each other using 1% DMSO as a baseline sample. Every treatment had four biological replicates except DOS where one replicate was excluded due to high background. All microarray data have been uploaded to GEO (GSE66812).

PCA analysis on conditions was used to determine similarity between samples using GeneSpring 12.6.1 GA-PA (Agilent) software. The microarray data have been uploaded to GEO: Accession GSE66812.

### RT-PCR Validation of Microarray Data

The StepOne Plus Real-Time PCR System (Applied Biosystems, Burlington, ON) was used to determine relative quantities of mRNA to validate findings from the microarray experiment. Using the QuantiTect Primer Assay (see S2 Table, Qiagen) and Power SYBR Green RNA-to-CT 1-Step Kit (Applied Biosystems) 20ng of RNA was reverse transcribed with ArrayScript UP Reverse Transcriptase at for 48°C for 30 minutes. This step was followed by a 10 minute incubation at 95°C to activate AmpliTaq Gold DNA Polymerase. cDNA was amplified and quantified over 40 cycles that each consisted of a denaturing step at 95°C for 15 seconds, an annealing step at 55°C for 30 seconds, and an elongation step at 72°C for 30 seconds. SYBRGreen fluorescence was quantified during the elongation step. A continuous melt curve from 60°C–95°C with 1% temperature increments was used to detect non-specific amplification to ensure accurate transcript quantification. All samples were run in triplicate with five biological replicates. *Hprt* was validated and used as a housekeeping gene to normalize starting RNA quantities. Relative expression was quantified using the ΔΔCt method. A reference sample was generated from a mixture of RNA from one biological replicate of all experimental conditions. Validated genes were selected based on relevant biological function, pathways, or fold changes found in the microarray datasets.

### Pathway Analysis

Probes within the 20-100th percentile after normalization, and registered as detected in at least one of two experimental conditions (control or treatment), were kept for analysis. Significance from this reduced list of entities was determined using a moderated t-test with Benjamini-Hochberg FDR correction and an asymptotic p-value computation. Probes with a fold change greater than 1.5 relative to controls and statistically significant were exported to Ingenuity Pathway Analysis v. 21249400 Sept 2014 (Qiagen, Redwood City, CA).

### Statistics

For cell viability assays, families of compounds were studied in independent groups of 5–6 compounds (see S1 Table for grouping). Each group shared a common control of either 0.4% or 1.0% DMSO. For each group two statistical analyses were done. For the high concentration (10^-4^M) significance was determined by one-way ANOVA (factor being compound) followed by Dunnett’s correction for multiple comparisons. This group usually contained 5–6 family comparisons. For lower concentrations, two-way ANOVA (factors being compound and concentration) followed by Dunnett’s multiple comparison test was used to determine significance. In this second case, a family was comprised of 20–24 comparisons. This method of analysis was selected due to the shared control DMSO sample. For qPCR experiments, one-way ANOVA corrected by Dunnett’s multiple comparison test was used for significance. All statistical analyses were computed using GraphPad Prism 6.05 (GraphPad Software, La Jolla, CA).

## Results

### Cell Viability

**Fig 2:**
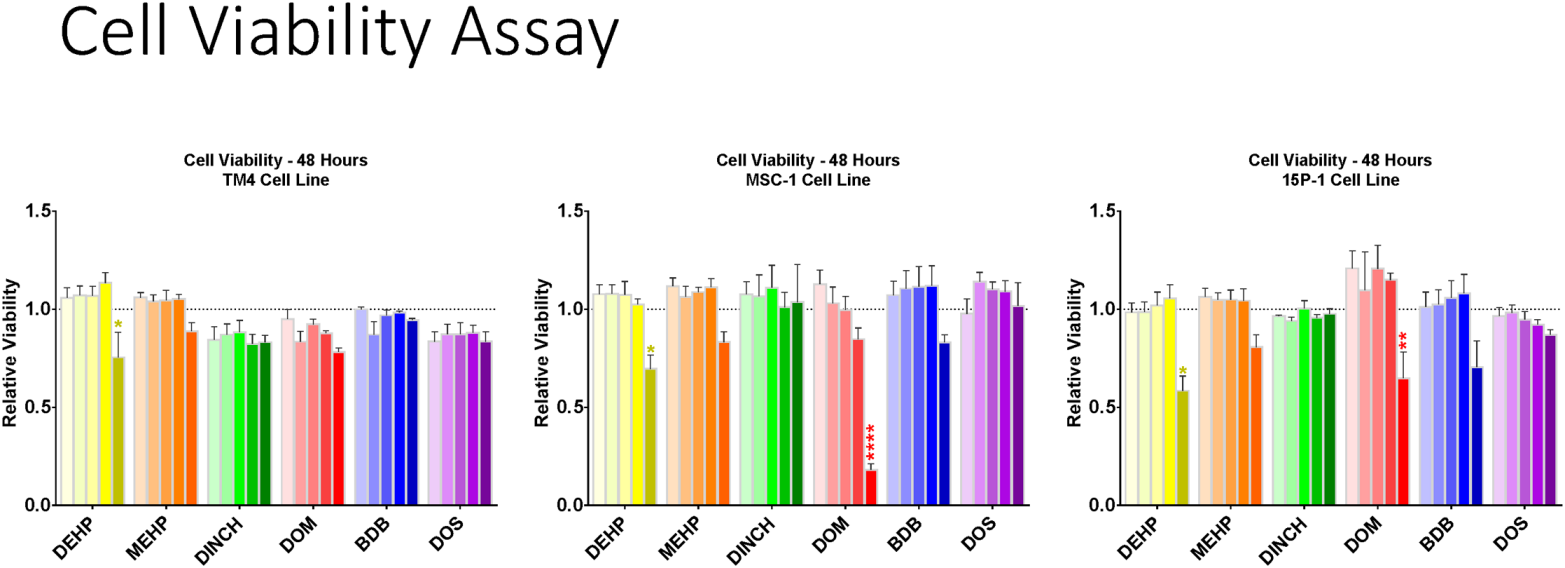
Cell viability following 48hr treatment. Values are expressed as a ratio of either 0.4% or 1.0% DMSO control (see S1 and S2 Figs). Viability was measured by the colorimetric MTT assay using concentrations of 10^-8^, 10^-7^, 10^-6^, 10^-5^, 10^-4^M (lightest to darkest bars) in (a) TM4, (b) MSC-1, (c) 15P-1 immortalized Sertoli cell lines. * = p≤0.05; ** = p≤0.01; **** = p ≤ 0.0001; n = 3–5 plated in triplicate.

Using the MTT assay, several families of plasticizers were studied for their effects on cell viability (S1 and S2 Figs). Plasticizers in the succinate and dibenzoate families generally had the least effect on cell viability compared to control while maleates and fumarates greatly decreased viability in most cases. Based on both desirable plasticizing properties and biological impact on cell viability, BDB and DOS were selected as safe alternative plasticizers while DOM was selected as a positive control, i.e., a plasticizer predicted to be toxic. From the supplemental data, the candidates used for further screening are presented in Fig 2. DEHP significantly decreased viability in all three cell lines examined with the most prominent effect being a 40% decrease in the 15P-1 cell line (p≤0.05) at 10^-4^M. DOM significantly decreased cell viability in both MSC-1 and 15P-1 cell lines at 10^-4^M. MEHP, DBD, DOS, and DINCH treatments did not change cell viability in any Sertoli cell line tested. In order to understand the adaptive processes that take place in the absence of cell death, the TM4 cell line was selected for microarray studies.

### Principle Component Analysis

**Fig 3:**
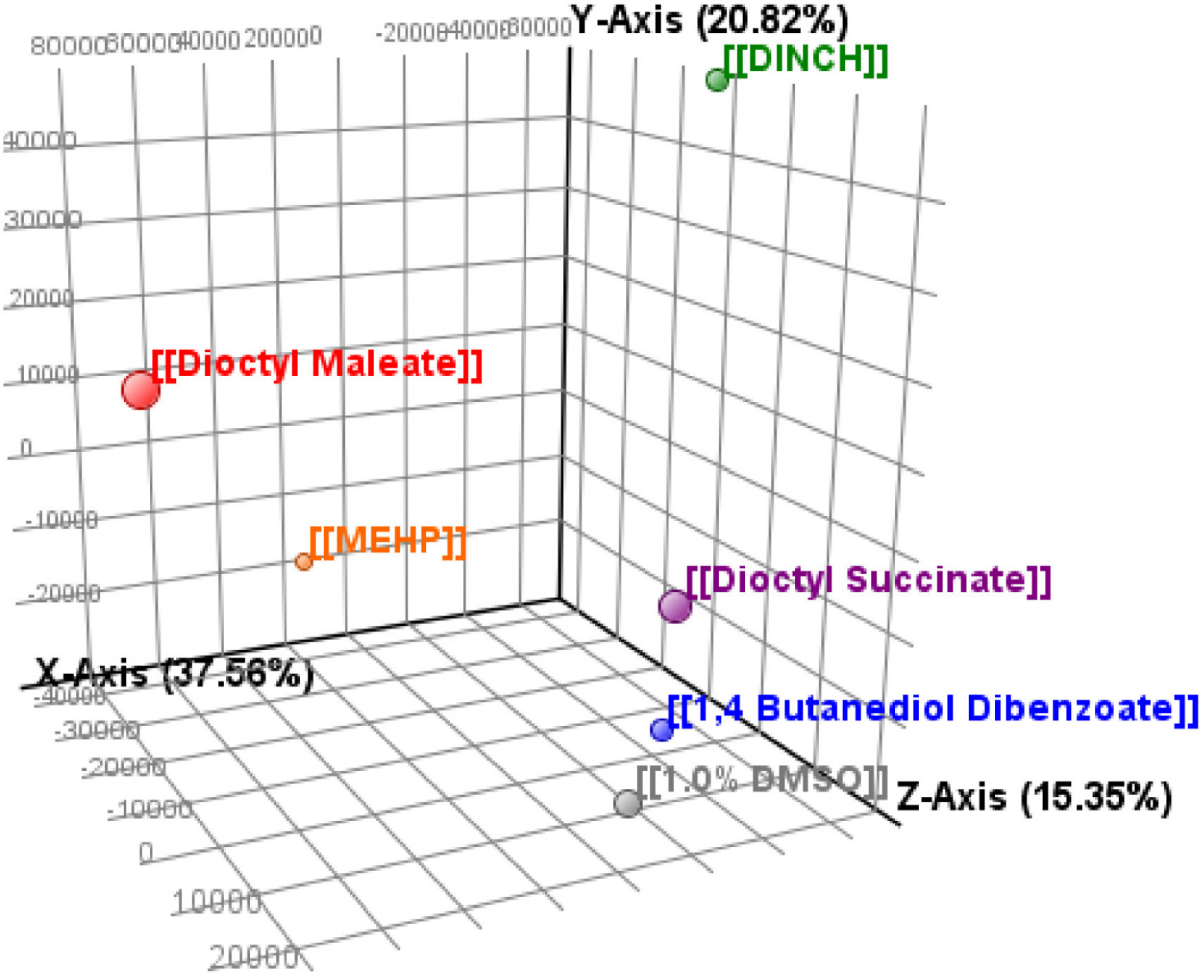
Principle component analysis of microarray data. TM4 Sertoli cells treated with 10^-4^M vehicle (DMSO), MEHP, DINCH, DOM, DOS or DBD for 48hr. n = 4 in all cases except DOS where n = 3.

In order to determine the overall gene transcript relationships in TM4 cells after treatment, a principle component analysis was done. This analysis is a mathematical algorithm that uses variation in datasets to determine principle components. Sample variance is plotted in three-dimensional space with similar treatments in close proximity (Fig 3). PCA analysis determined three major components representing 37.56%, 20.82% and 15.35% of variance between all samples. BDB and DOS clustered closely to the control sample with vector magnitudes of 23,703 and 29,931 units respectively from the 1.0% DMSO sample. In comparison, MEHP (63,217), DINCH (97,601), and DOM (122,994) all clustered further away from control.

### Differential Gene Expression

**Fig 4:**
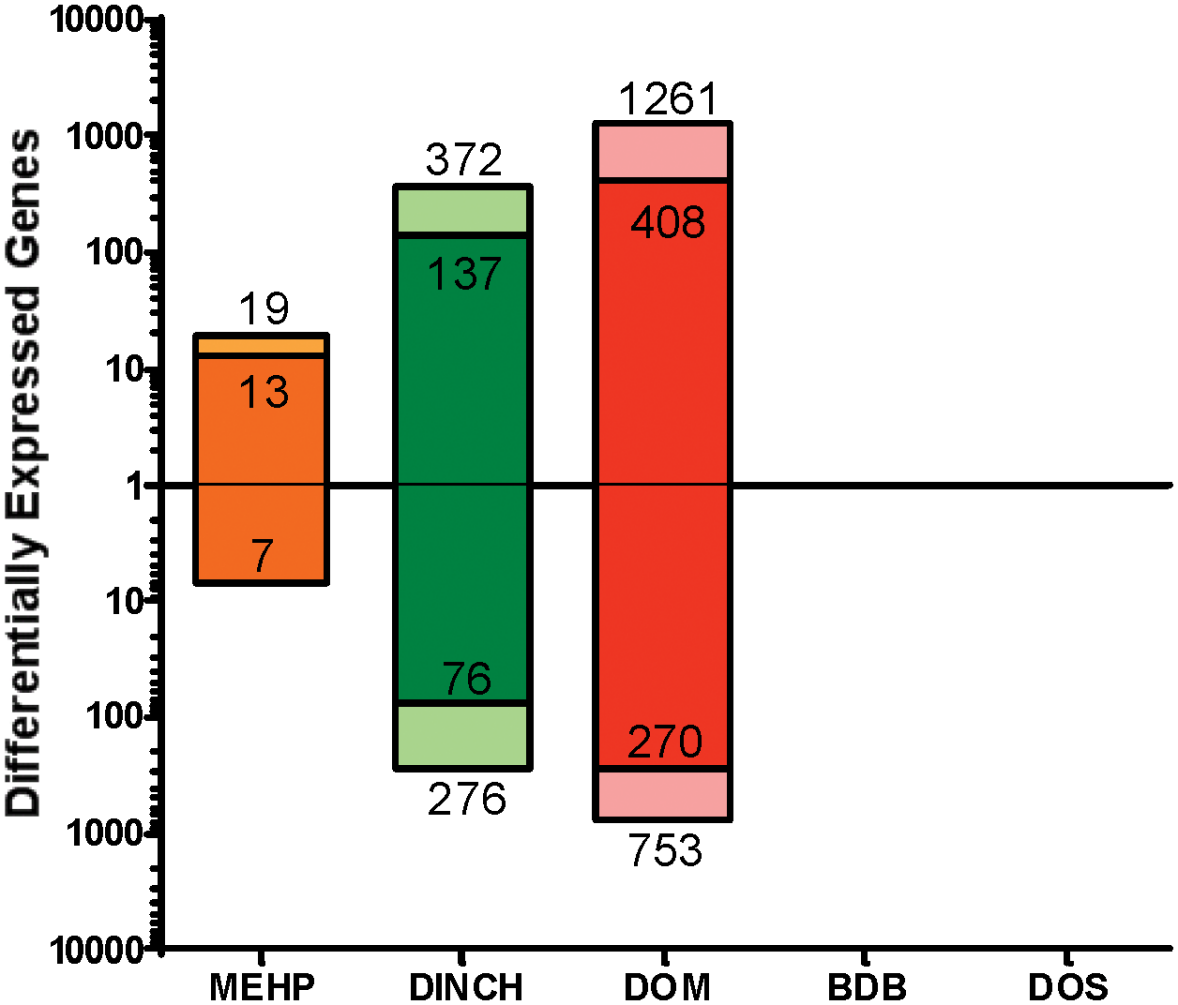
The number of uniquely mapped genes significantly changed following 48hr treatment of TM4 Sertoli cells. Saturated color (inner bar) indicates unique mapped genes that were significantly changed by >2.0 fold determined by moderated t-test and Benjamini-Hochberg FDR correction (P>0.05) while lighter bars represent genes that were changed by > 1.5 fold with the same statistical criteria.

**Fig 5:**
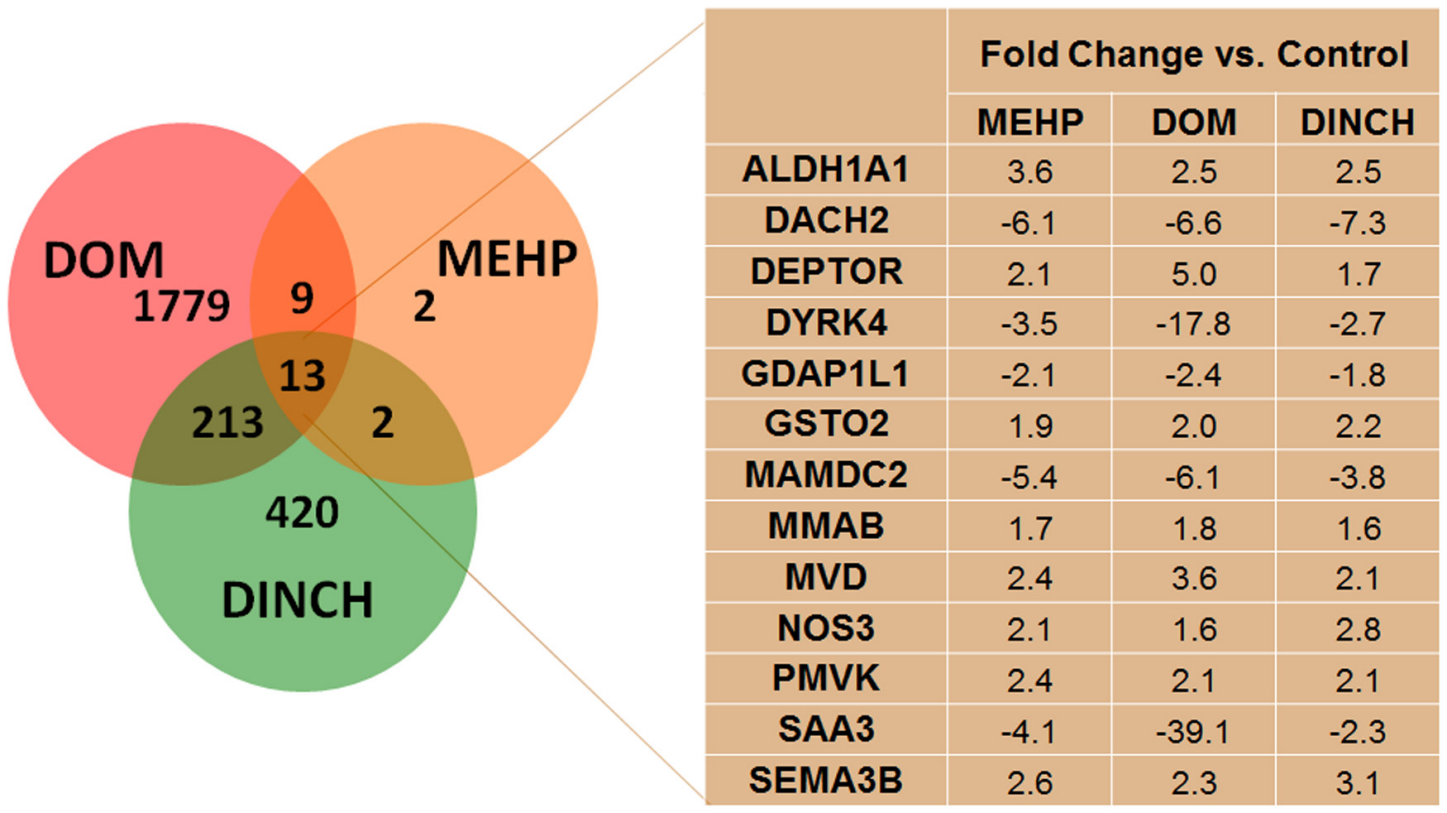
Commonalities in differential gene expression following treatment of TM4 Sertoli cells for 48 hours. Left panel: Venn diagram of distinct and common genes between DINCH, MEHP and DOM treatment groups (10^-4^M) with changes in expression greater than 1.5 fold. Right panel: Table showing 13 genes common to the three treatments and the fold changes in response to treatment.

DOS and BDB did not have any differentially expressed genes at 10^-4^M after statistical corrections were applied relative to the 1.0% DMSO control (Fig 4). MEHP had relatively few genes changed, while DINCH had 1,261 uniquely mapped genes up-regulated and 753 down-regulated by 1.5 fold or greater. DOM had 2,014 differentially expressed genes with the largest overall magnitude fold-changes compared to MEHP and DINCH. Almost a third (226/648) of the genes significantly changed after DINCH treatment overlapped with those changed after treatment with DOM (Fig 5).

**Fig 6:**
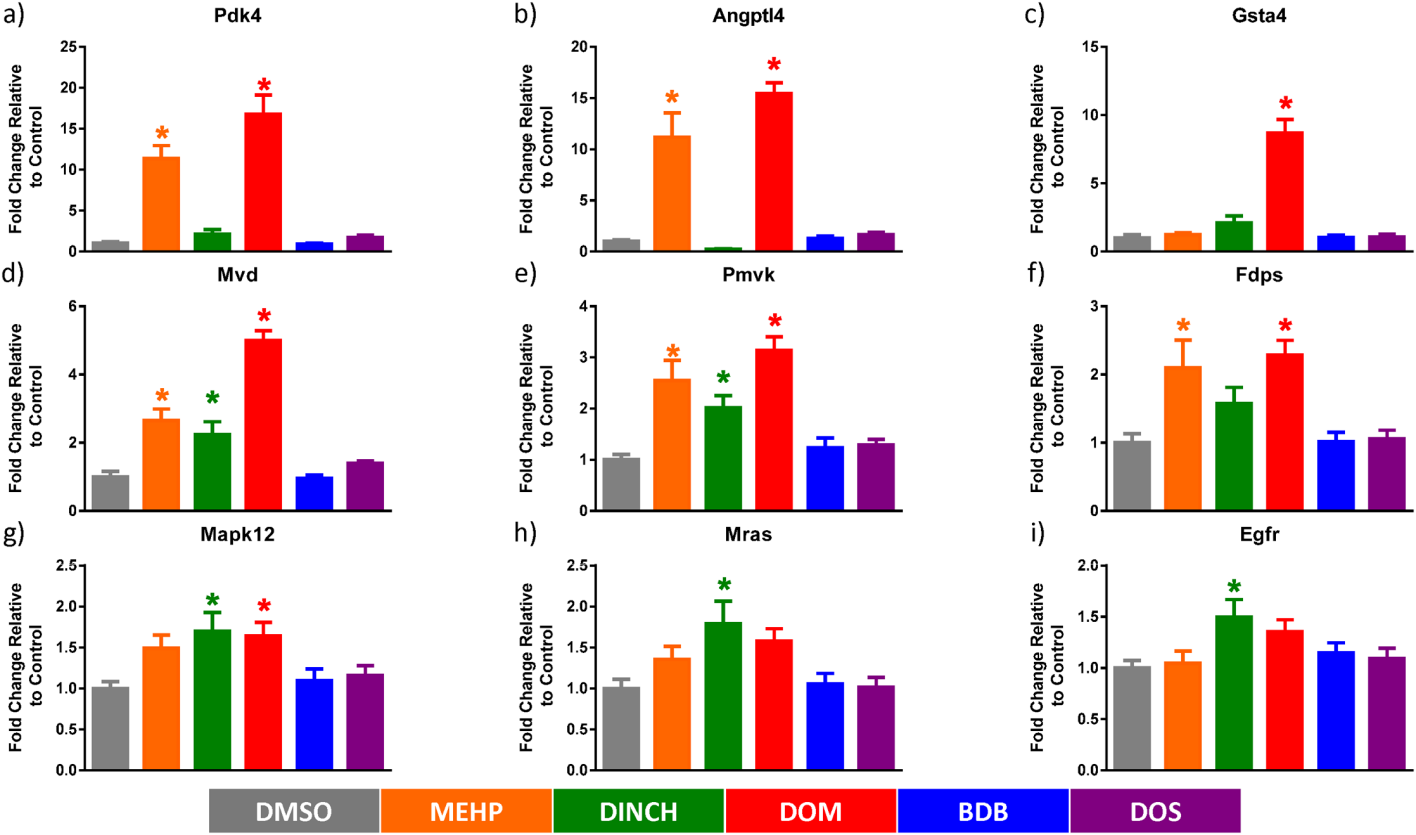
Gene expression quantification by qPCR for selected transcripts after treatment of TM4 cells with 10^-4^M plasticizers. Values are normalized to the DMSO control sample which was set to 1. Graphs have been organized by the magnitude of the scale of the axis. Significance was determined by one-way ANOVA corrected by Dunnett’s multiple comparison test. n = 4–5 biological replicates plated in triplicate. * P<0.05

Thirteen genes were differentially regulated after MEHP, DINCH and DOM treatments. Of these shared genes, *Mvd* and *Pmvk* were validated by qPCR (Fig 6d and 6e) because of their involvement in cholesterol biosynthesis. These genes are also downstream targets of SREBF2 [49], which is a transcription factor predicted to be activated by MEHP treatment in our dataset (z-score = 2.0; p≤2.48e-6), and has an important role in cholesterol homeostasis. In order to further verify our model, *Pdk4* and *Angptl4* were validated (Fig 6a and 6b) as both are downstream targets of PPAR, and Pdk4 is known to be upregulated following treatment with MEHP [50]. All four genes were significantly upregulated after treatment with both MEHP and DOM.

### Pathway Analysis

To understand the biological relationship of differentially expressed genes, those genes that were significantly changed by 1.5 fold or more were imported into Ingenuity Pathway Analysis. The MEHP dataset was enriched for genes involved in cholesterol biosynthesis and nuclear receptor signalling pathways (Table 1). The DOM dataset was downregulated for genes involved in DNA replication, folate metabolism, and cell-cycle progression, and upregulated for genes involved in xenobiotic metabolism, aryl hydrocarbon signalling, glutathione mediated detoxification, oxidative stress responses, and cholesterol biosynthesis (Table 2). Findings were confirmed by qPCR by validating *Gsta4* and *Fdps* (Fig 6c and 6f) in addition to other targets previously mentioned. In addition to the oxidative stress and cholesterol biosynthesis pathways previously observed, treatment with DINCH generally upregulated genes involved in ERK/MAPK, Ephrin, and Rho signalling pathways (Table 3). Furthermore, many of these pathways were assigned z-scores above 2 by the pathway analysis software, suggesting that based on the directionality of our gene expression dataset, these pathways are predicted to be activated. These findings were confirmed by qPCR for *Mapk12*, *Mras*, and *Egfr* (Fig 6g, 6h and 6i). Tables 1, 2 and 3 show selected pathways that were significantly enriched in our dataset and genes that were differentially regulated by 1.5 fold or greater. More details regarding the proportion of matching entities, the log (p-value), and the directionality of the response for each plasticizer can be found in the appropriate tables.

**Table 1:** Pathway Analysis of 10^-4^M MEHP gene expression in TM4 cells^1^.

Pathway	-log(p-value)	Ratio	z-score	Upregulated	Down-regulated
PXR/RXR Activation	2.46	2/59 (0.04)	N/A	*Aldh1a1*, *Cpt1a*	
RAR Activation	2.69	3/172 (0.02)	N/A	*Aldh1a1*, *Tgfb2*	*Dhrs3*
Aryl Hydrocarbon Receptor Signaling	3.04	3/130 (0.03)	N/A	*Aldh1a1*, *Tgfb2*, *Gsto2*	
Superpathway of Cholesterol Biosynthesis	9.43	5/27 (0.19)	N/A	*Mvd*, *Nsdhl*, *Pmvk*, *Acat2*, *Lss*	

^1^The tables list pathways deemed to be significantly enriched in each dataset according to Ingenuity Pathway Analysis. The first column is the curated name of the pathway. The second column is the −log(p-value) associated with this pathway determined by the software using a Fisher’s exact test. The ratio column is the number of entities in a dataset that match the entities in the curated pathway with the value in brackets representing the ratio. The z-score is a value assigned by the software to indicate whether a pathway is predicted to be activated or repressed when sufficient data is available. The last two columns mention genes that are either up- or down-regulated in our dataset that are also members of the curated pathway.

**Table 2:** Pathway Analysis of 10^-4^M DOM gene expression in TM4 cells^1^.

Pathway	-log(p-value)	Ratio	z-score	Upregulated	Down-regulated
PXR/RXR Activation	2.46	2/59 (0.04)	N/A	*Aldh1a1*, *Cpt1a*	
Mismatch Repair in Eukaryotes	1.74	5/16 (0.32)	N/A		*Pcna*, *Rfc4*, *Msh3*, *Fen1*, *Rfc5*
VDR/RXR Activation	1.95	15/78 (0.2)	-1.414	*Foxo1*, *Runx2*, *Hoxa10*, *Ncor1*, *Sema3b*, *Cst6*, *Prkd3*, *Prkca*	*Bglap*, *Spp1*, *Ccnc*, *Ccl5*, *Hes1*, *Klf4*, *Cxcl10*,
Cyclins and Cell Cycle Regulation	2.5	16/75 (0.22)	-2.53	*Hdac9*, *Abl1*, *Ccnb2*, *Hdac5*, *Tgfb1*, *Ppm1l*, *Tgfb3*, *Btrc*, *Gsk3b*	*Pa2g4*, *Suv39h1*, *Cdkn2b*, *Ccnb1*, *Cdkn2d*, *Ccne1*, *E2f2*
Xenobiotic Metabolism Signaling	2.96	40/239 (0.17)	N/A	*Aldh4a1*, *Ftl*, *Gstm5*, *Ugt1a6*, *Gsta5*, *Arnt*, *Hmox1*, *Aldh1a1*, *Gstm3*, *Ppm1l*, *Gstm4*, *Chst3*, *Chst11*, *Aldh3a1*, *Prkd3*, *Cited2*, *Aldh6a1*, *Gstk1*, *Camk2b*, *Prkca*, *Gstm1*, *Mgst1*, *Map3k6*, *Gstm3*, *Nqo1*, *Hdac5*, *Esd*, *Pik3r3*, *Mgst2*, *Cat*, *Aldh3b1*, *Map3k8*, *Gsto2*, *Mgst3*,	*Ndst3 Chst7*, *Camk2d*, *Rras2*, *Aldh1l2*, *Aldh18a1*
Folate Transformations I	3.02	5/9 (0.56)	N/A	*Mthfs*	*Mthfd1l*, *Shmt1*, *Mthfd1*, *Shmt2*
LPS/IL-1 Mediated Inhibition of RXR Function	3.13	35/197 (0.18)	-0.333	*Aldh4a1*, *Gstm5*, *Gsta5*, *Alas1*, *Abcb9*, *Aldh1a1*, *Scarb1*, *Gstm3*, *Chst3*, *Gstm4*, *Xpo1*, *Chst11*, *Cpt1c*, *Aldh3a1*, *Hmgcs1*, *Aldh6a1*, *Gstk1*, *Gstm1*, *Mgst1*, *Cpt1a*, *Gstm3*, *Ly96*, *Mgst2*, *Cat*, *Aldh3b1*, *Gsto2*, *Acox3*, *Mgst3*	*Ppargc1b*, *Ndst3*, *Apoe*, *Chst7*, *Aldh1l2*, *Aldh18a1*, *Acsl1*
Aryl Hydrocarbon Receptor Signaling	4.02	28/130 (0.22)	0.333	*Aldh4a1*, *Gstm5*, *Gsta5*, *Arnt*, *Ctsd*, *Aldh1a1*, *Tgfb1*, *Gstm3*, *Gstm4*, *Aldh3a1*, *Aldh6a1*, *Gstk1*, *Gstm1*, *Mgst1*, *Gstm3*, *Nqo1*, *Mgst2*, *Tgfb3*, *Aldh3b1*, *Gsto2*, *Esr1*, *Mgst3*	*Mcm7*, *Pola1*, *Rbl1*, *Ccne1*, *Aldh1l2*, *Aldh18a1*
Cell Cycle: G1/S Checkpoint Regulation	4.07	17/61 (0.28)	0.277	*Hdac9*, *Smad3*, *Abl1*, *Hdac5*, *Cdkn2d*, *Foxo1*, *Tgfb1*, *Tgfb3*, *Btrc*, *Gsk3b*	*Pa2g4*, *Suv39h1*, *Rbl1*, *Cdkn2b*, *Ccne1*, *Gnl3*, *E2f2*
NRF2-mediated Oxidative Stress Response	4.54	35/168 (0.21)	1.5	*Ftl*, *Gstm5*, *Gsta5*, *Dnajb2*, *Dnaja1*, *Hmox1*, *Scarb1*, *Gstm3*, *Abcc1*, *Gstm4*, *Gclm*, *Gsk3b*, *Prkd3*, *Gstk1*, *Prkca*, *Gstm1*, *Mgst1*, *Gstm3*, *Nqo1*, *Dnajb14*, *Bach1*, *Pik3r3*, *Mgst2*, *Cat*, *Dnajc14*, *Aox1*, *Gsto2*, *Ptplad1*, *Enc1*, *Mgst3*, *Ephx1*	*Pmf1*, *Atf4*, *Herpud1*, *Rras2*,
Cell Cycle Control of Chromosomal Replication	4.67	11/26 (0.43)	N/A		*MCM5*, *MCM3*, *ORC2*, *RPA3*, *MCM2*, *CDT1*, *CDC6*, *ORC6*, *MCM4*, *MCM7*, *RPA2*
tRNA Charging	4.96	14/38 (0.37)	N/A	*Farsa*	*Cars*, *Cars2*, *Mars2*, *Gars*, *Tars*, *Farsb*, *Nars*, *Lars*, *Wars*, *Rars*, *Aars*, *Sars*, *Iars*
Glutathione-mediated Detoxification	6.26	12/23 (0.53)	N/A	*Gstm1*, *Mgst1*, *Mgst2*, *Gstm5*, *Gstm3*, *Gstm3*, *Gsta5*, *Gstm4*, *Gsto2*, *Gsta4*, *Mgst3*, *Gstk1*	
Superpathway of Cholesterol Biosynthesis	14.3	20/27 (0.75)	N/A	*Mvd*, *Sqle*, *Nsdhl*, *Pmvk*, *Acat2*, *Idi1*, *Mvk*, *Hsd17b7*, *Msmo1*, *Tm7sf2*, *Sc5d*, *Ggps1*, *Fdps*, *Fdft1*, *Dhcr7*, *Dhcr24*, *Lss*, *Hmgcr*, *Hmgcs1*, *Cyp51a1*	

^**1**^ See footnote to Table 1 for explanation of column headings

**Table 3:** Pathway Analysis of 10-4M DINCH gene expression in TM4 cells^1^.

Pathway	-log(p-value)	Ratio	z-score	Upregulated	Down-regulated
NRF2-mediated Oxidative Stress Response	1.3	10/168 (0.06)	1	*Ftl*, *Gstm5*, *Rras*, *Nqo1*, *Mras*, *Dnajb2*, *Gsto2*	*Abcc4*, *Dnajc11*, *Fos*
RhoA Signaling	1.83	9/117 (0.08)	2.333	*Sept8*, *Ngef*, *Rhpn2*, *Ptk2b*, *Cdc42ep5*, *Gna13*, *Sept6*, *Pkn1*	*Myl4*
VDR/RXR Activation	1.85	7/78 (0.09)	1	*Cxcl10*, *Serpinb1*, *Tgfb2*, *Sema3b*, *Ccl5*, *Rxra*	*Ccnc*
ERK/MAPK Signaling	1.87	12/176 (0.07)	2.714	*Ptk2b*, *Rras*, *Hspb2*, *Mras*, *Prkar1b*, *Rps6ka5*, *Rapgef3*, *Creb3l4*, *Rps6ka1*	*Fos*, *Ywhag*, *Ppp1r7*
Superpathway of Cholesterol Biosynthesis	1.96	4/27 (0.15)	N/A	*Mvd*, *Fdps*, *Dhcr7*, *Pmvk*	
p38 MAPK Signaling	2.01	9/109 (0.09)	2.646	*Cdc25b*, *Tifa*, *Hspb2*, *Tgfb2*, *Map4k1*, *Rps6ka5*, *Creb3l4*, *Rps6ka1*, *Mapk12*	
Signaling by Rho Family GTPases	2.09	15/227 (0.07)	2.496	*Sept8*, *Arhgef4*, *Ptk2b*, *Cdc42ep5*, *Mapk12*, *Pkn1*, *Arfip2*, *Mras*, *Gna13*, *Sept6*, *Arhgef9*, *Cdh13*	*Gnaq*, *Fos*, *Myl4*
Glutathione-mediated Detoxification	2.21	4/23 (0.18)	N/A	*Gstz1*, *Gstm5*, *Gsto2*, *Gstt1*	
Ephrin Receptor Signaling	2.81	14/171 (0.09)	N/A	*Ngef*, *Rras*, *Sh2d3c*, *Vegfb*, *Vegfc*, *Creb3l4*, *Mras*, *Figf*, *Gna13*	*Itsn1*, *Gnaq*, *Efnb2*, *Sdcbp*, *Efnb3*
ERK5 Signaling	3.75	9/62 (0.15)	1.414	*Rras*, *Mras*, *Rps6ka5*, *Creb3l4*, *Gna13*, *Rps6ka1*	*Fos*, *Ywhag*, *Gnaq*

^**1**^ See footnote to Table 1 for explanation of column headings

## Discussion

Despite the large body of evidence indicating that several phthalates such as DEHP can be toxic, regulatory agencies have only recently implemented regulations that limit their use. Our study provides evidence supporting the role of PPAR in mediating phthalate toxicity. While this is the first report of gene expression changes in the TM4 Sertoli cell line following treatment with MEHP, our findings of gene activation downstream of PPAR, lipid metabolism, and nuclear receptor involvement support observations from other cell types and animal models [17, 18, 44, 51], further supporting a role for *in vitro* toxicogenomic screening as a model to predict *in vivo* toxicity. DEHP decreased cell viability in multiple cell lines as previously reported in MA-10 Leydig cells after 48 hour treatment [52]. While DEHP is not usually considered to be biologically active, Sertoli cells express a hormone sensitive lipase that is capable of metabolizing DEHP into biologically active or toxic metabolites [53, 54]. Previous studies have shown that 2-ethylhexanal, a by-product of DEHP metabolism, can decrease cell viability [52], but we are unable to determine whether DEHP was metabolised in our model or whether DEHP or one of its metabolites other than MEHP was responsible for the observed decrease in viability. Taken together, our results for DEHP and MEHP are consistent with previous findings.

While DOM is a good plasticizer, the accumulation of the toxic metabolite monooctyl maleate in the presence of the common soil bacterium *Rhodococcus rhodocrous* makes DOM a less desirable alternative [39]. In our studies, the maleate family generally decreased cell viability in most cell lines. Diethyl maleate (DEM) is a well-characterized compound used to deplete glutathione and induce cell damage by increasing reactive oxygen species [55]. Few studies have used DOM in a biological context, but DOM and DEM share a common maleate core that conjugates to glutathione in a reaction that is catalyzed by glutathione S-transferases (GST) [56]. Furthermore it has been reported that inducers of GST gene expression are typically Michael acceptors which are compounds that contain an unsaturated bond with an electron withdrawing group [57]. Therefore, it is not surprising that DOM upregulated several GST isoforms and several genes involved in response to reactive oxygen species damage. Taken together, these data suggest DOM or similar maleate based plasticizers with varying side-chain lengths would not be suitable replacements for phthalate plasticizers based on the read-across principle [58].

DINCH production has grown to an annual capacity of 200,000 metric tons in 2013 [59]. While DINCH does not leach as much as DEHP from PVC [4], by-products of DINCH metabolism previously undetectable in human urine samples collected between 2000 and 2001, can be detected in samples collected from 2007–2012 in increasing quantities, indicating increasing human exposure [60]. Despite the marketing of this compound as a safe phthalate replacement, our microarray analysis suggests that DINCH is biologically active. Interestingly, 30% of genes that were differentially regulated by DINCH were also changed by DOM treatment, which had more pronounced effects on cell viability and gene expression. The DINCH gene expression dataset was enriched for genes involved in cellular movement, glutathione mediated detoxification, and important signalling pathways such as RhoA and ERK/MAPK. Many of these pathways were predicted to be activated, and can be correlated back to biological roles in Sertoli cell proliferation, differentiation, cytoskeleton, and junctional dynamics [61–63]. Whether these changes in gene expression are physiologically relevant or whether compensatory mechanisms can take place to maintain homeostasis at an organismal level remains to be determined.

Two novel plasticizers (BDB, DOS) did not have an effect on cell viability following 48 hour exposure *in vitro*. Furthermore, there was no significant change in gene expression following microarray analysis. While our data suggest that DOS and BDB are potential replacements for phthalate plasticizers, further testing is required to determine whether there is systemic toxicity after chronic or developmental exposure to these compounds.

In our study, we have proactively screened a larger list of candidate plasticizers to identify those least likely to have deleterious biological effects. In addition to being good plasticizers, dibenzoates without an ether function and succinates can easily biodegrade [38, 64]. Succinates have the added advantage in that they can be sourced from the by-products of fermentation [38]. Thus, unlike phthalate based compounds, succinic acid production does not depend on petroleum refinement; which will minimize the associated environmental impact and dependence on petroleum based products [65, 66]. Based on our findings and previously published results on their plasticizing properties, dioctyl succinate and 1,4 butanediol dibenzoate are promising replacements for DEHP and other phthalate based plasticizers.

## Supporting Information

S1 Fig Cell viability data determined by MTT assay in three distinct Sertoli cell lines. Figure shows results for phthalates (DEHP and bioactive metabolite MEHP), a commercial alternative plasticizer (DINCH), and a modified dibenzoate series. Results for other alternative plasticizers can be found in S2 Fig.
(TIF)

S2 Fig Cell viability data determined by MTT assay in three distinct Sertoli cell lines. Figure shows results for succinate, maleate, and fumarate plasticizers.
(TIF)

S1 Table List of all plasticizers used in this study with names, abbreviations, CAS numbers and source. Grouping analysis refers to which compounds were tested together on the same 96-well plate for the MTT assay (and therefore share common control DMSO treated samples).
(TIF)

S2 Table List of primers used for RT-PCR validation. Table includes catalogue number, catalogue name, gene name, transcript reference number, and length of the aplified PCR product.
(TIF)
