# Supplementary figures and images for: Toxicogenomic Screening of Replacements for Di(2-Ethylhexyl) Phthalate (DEHP) Using the Immortalized TM4 Sertoli Cell Line
Source: PLoS One. 2015 Oct 7;10(10):e0138421. doi: 10.1371/journal.pone.0138421 (PMC4596883; doi:10.1371/journal.pone.0138421)

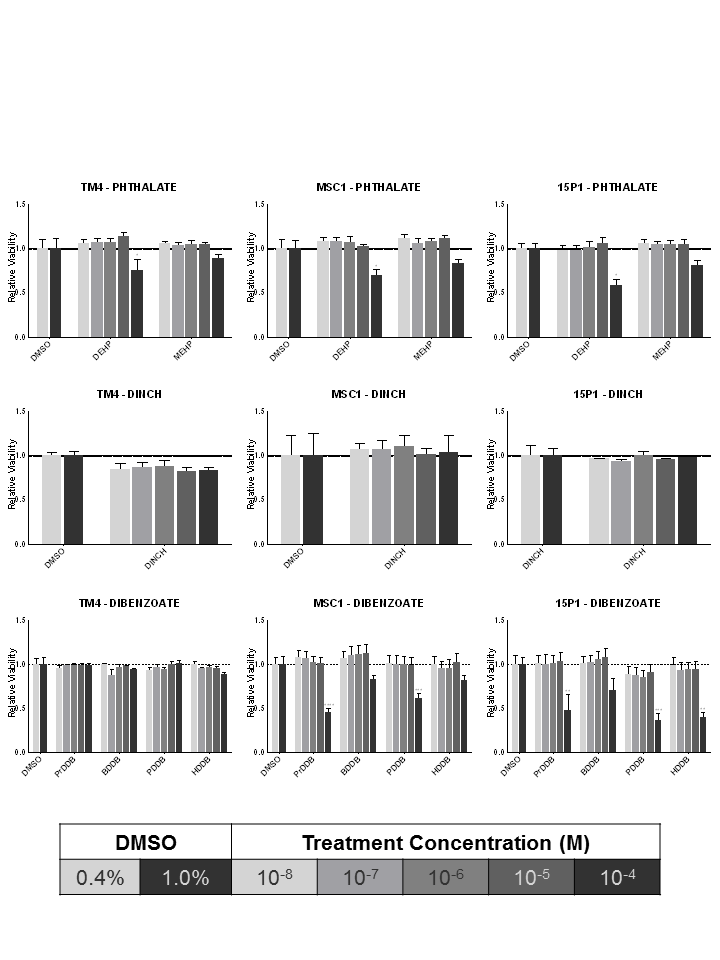

Supplement: S1 Fig — Figure shows results for phthalates (DEHP and bioactive metabolite MEHP), a commercial alternative plasticizer (DINCH), and a modified dibenzoate series. Results for other alternative plasticizers can be found in S2 Fig. (TIF) [file pone.0138421.s001.tif]

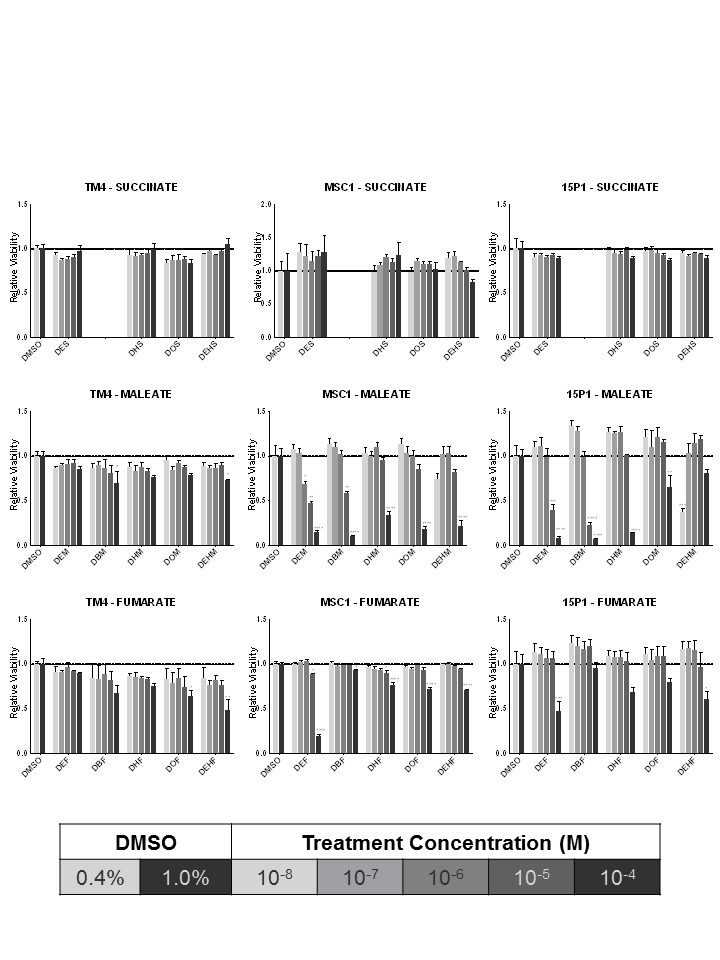

Supplement: S2 Fig — Figure shows results for succinate, maleate, and fumarate plasticizers. (TIF) [file pone.0138421.s002.tif]

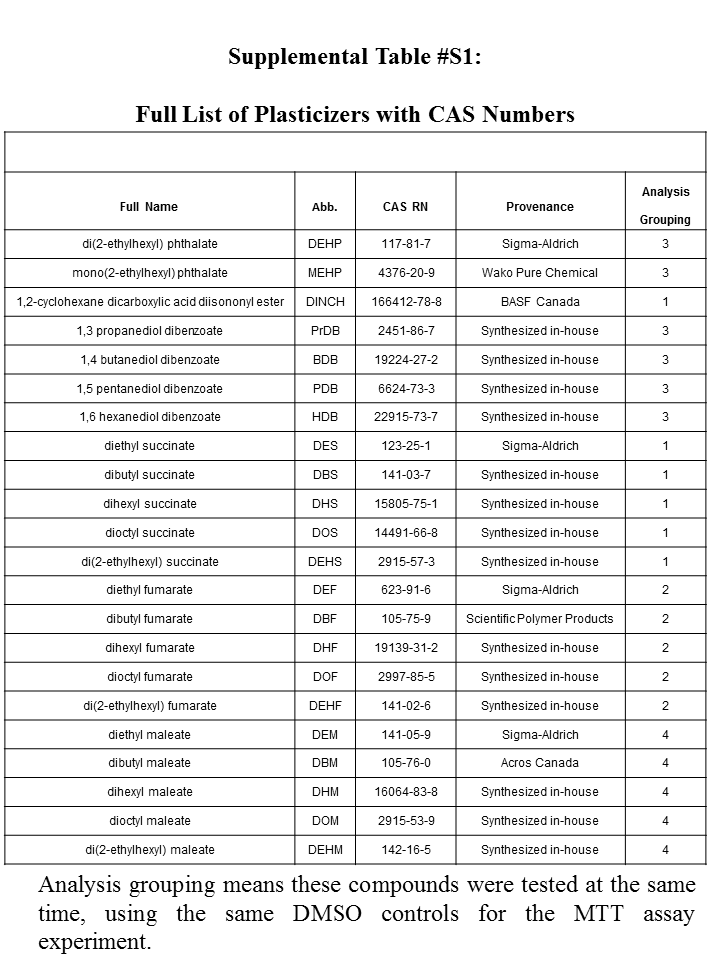

Supplement: S1 Table — Grouping analysis refers to which compounds were tested together on the same 96-well plate for the MTT assay (and therefore share common control DMSO treated samples). (TIF) [file pone.0138421.s003.tif]

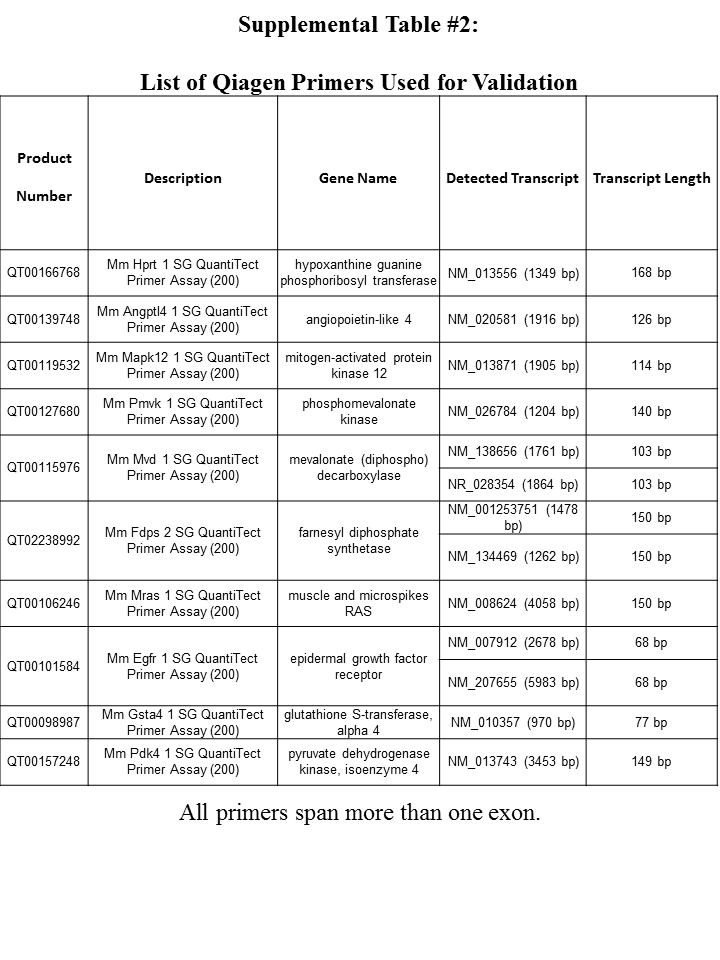

Supplement: S2 Table — Table includes catalogue number, catalogue name, gene name, transcript reference number, and length of the aplified PCR product. (TIF) [file pone.0138421.s004.tif]
